# Economic Evaluation of a Hybrid Desalination System Combining Forward and Reverse Osmosis

**DOI:** 10.3390/membranes6010003

**Published:** 2015-12-29

**Authors:** Yongjun Choi, Hyeongrak Cho, Yonghyun Shin, Yongsun Jang, Sangho Lee

**Affiliations:** School of Civil and Environmental Engineering, Kookmin University, Jeongneung-Dong, Seongbuk-Gu, Seoul 136-702, Republic of Korea; choiyj1041@gmail.com (Y.C.); clarkcho@kookmin.ac.kr (H.C.); shinyonghyun@kookmin.ac.kr (Y.S.); cutymonkey5@naver.com (Y.J.)

**Keywords:** FO-RO hybrid, FO, cost, modeling

## Abstract

This study seeks to evaluate the performance and economic feasibility of the forward osmosis (FO)–reverse osmosis (RO) hybrid process; to propose a guideline by which this hybrid process might be more price-competitive in the field. A solution-diffusion model modified with film theory was applied to analyze the effects of concentration polarization, water, and salt transport coefficient on flux, recovery, seawater concentration, and treated wastewater of the FO process of an FO-RO hybrid system. A simple cost model was applied to analyze the effects of flux; recovery of the FO process; energy; and membrane cost on the FO-RO hybrid process. The simulation results showed that the water transport coefficient and internal concentration polarization resistance are very important factors that affect performance in the FO process; however; the effect of the salt transport coefficient does not seem to be large. It was also found that the flux and recovery of the FO process, the FO membrane, and the electricity cost are very important factors that influence the water cost of an FO-RO hybrid system. This hybrid system can be price-competitive with RO systems when its recovery rate is very high, the flux and the membrane cost of the FO are similar to those of the RO, and the electricity cost is expensive. The most important thing in commercializing the FO process is enhancing performance (e.g.; flux and the recovery of FO membranes).

## 1. Introduction

A seawater desalination process separates seawater into two streams—namely, a fresh water stream containing a low concentration of dissolved salts, and a concentrated brine stream [1]. This desalination process requires some form of energy, and it utilizes several different technologies in separation. Many desalination technologies have been developed over the years on the basis of thermal distillation, membrane separation, freezing, and electro-dialysis [2]. Since 2005 in particular, growing interest has been observed in osmotically driven membrane processes and, more specifically, in forward osmosis (FO), which has been evaluated as a promising alternative to reverse osmosis (RO) for seawater desalination, to produce fresh water while consuming low levels of energy [3]. FO uses a concentrated draw solution to generate high osmotic pressure, which pulls water through a semi-permeable membrane from the feed water. The semi-permeable membrane acts as a barrier that allows small molecules such as those of water to pass through, while larger molecules—such as those of salts, sugars, proteins, viruses, and bacteria—are “sifted” out. The draw solute is then separated from the diluted draw solution, to recycle the solute and produce clean product water [4,5]. In the context of seawater desalination, FO typically involves a two-step process. FO can be implemented as a pre-treatment for RO [6]; in this case, low-salinity water is used as an FO feed solution to dilute seawater. As a result of using seawater dilution, less energy is required to produce fresh water, given the lower osmotic pressure of the feed. This type of scheme is referred to as an FO-RO hybrid [7,8]; it is typically of interest when impaired water is used as feed, as the scheme can uniquely combine water reuse and desalination [8,9]. The first industrial FO desalination system in Oman was inaugurated in September 2012, testifying to the potential future of this technology [2]. However, FO processes are still nascent in terms of commercial applications [1]; therefore, further work is still required to bring FO technologies into practice, including the development of new FO membranes, the design of new hybrid systems, the development of new draw salts, and the creation of recovery systems [3].

This study seeks to evaluate the performance and economic feasibility of the FO-RO hybrid process, and hence to propose guidelines by which the hybrid process could become more price-competitive. We developed a model to predict the performance of this hybrid system, which leverages both FO and RO; this model is based on the solution-diffusion model, with modifications from film theory. The effects of external and internal concentration polarization (ECP and ICP) on FO efficiency are also considered in the model. A simple cost model was applied to analyze the effects of flux, recovery, membrane, and energy cost on the FO-RO hybrid process.

## 2. Models

We applied a solution-diffusion model modified with film theory model to analyze the performance of the FO-RO process. To analyze the effects of major parameters such as flux, recovery, membrane, and energy cost on RO and FO-RO hybrid process, we formulated simple cost functions. Numerous reports analyzing the cost of RO processes have been published, and so there exist many and various cost functions [10,11,12,13,14,15]. From them, we drew as broad a range of cost functions as possible.

### 2.1. Reverse Osmosis Model

For an RO system, the water flux (*J_w_*) and solute flux (*J_s_*) equations can be defined as follows [16]:

(1) $$J_{w}=A(P-\Delta \pi_{C_{F,m}})$$

(2) $$J_{s}=B(C_{F,m}-C_{p}),$$

where *A* is the water transport coefficient, *B* is the salt transport coefficient, *C_F,m_* is the salt concentration on the membrane surface, *C_p_* is the salt concentration on the permeate side, *Δπ* is the osmotic pressure, and *P* is the feed pressure.

*C_F,m_* is calculated according to film theory, so that we can interpret the concentration polarization; the solvent concentration profile on the surface can be calculated according to the following equation [17].

(3) $$\frac{C_{F,m}-C_{p}}{C_{F,b}-C_{p}}=e^{\frac{J_{w}}{k}},$$

where *C_F,b_* is the salt concentration in the bulk solution, *k* is the mass transfer coefficient for the back diffusion of the solute from the membrane to the bulk solution on the high-pressure (HP) side of the membrane [17,18], as follows.

(4) $$k=\frac{ShD}{d_{h}}$$

(5) $$Sh=1.85{\left(Re\;Sc\frac{d_{h}}{L}\right)}^{0.33}\;(Re\;\leq \;2100)$$

(6) $$Sh=0.04{Re}^{0.75}Sc^{0.33}\;(Re\;>\;2100)$$

where *D* is the diffusion coefficient, *d_h_* is the hydraulic diameter, *Sh* is the Sherwood number, *Re* is the Reynolds number, and *Sc* is the Schmidt number.

**Figure 1:**
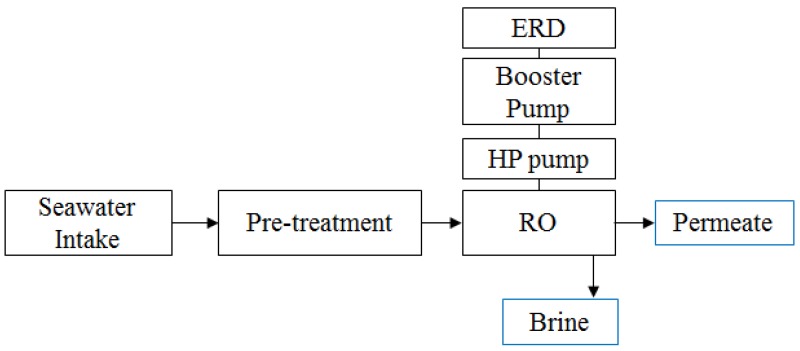
Schematic diagram of reverse osmosis (RO) system, used to evaluate economic feasibility.

The RO system for economic evaluation is made of six major parts—namely, the seawater intake, pre-treatment process, HP pump, booster pump, RO membrane module, and energy recovery device (ERD), as shown as Figure 1.

The capital and operating costs (CC and OC) of the intake, pre-treatment process, HP pump, booster pump, and ERD are expressed as follows [12,13,14,15].

(7) $$CC_{IT\_RO}[\$]=598\times {\left(Q_{f}[m^{3}/d]/0.9\right)}^{0.78}$$

(8) $$OC_{IT\_RO}[\$/d]=0.028P_{IT}[bar]Q_{f}[m^{3}/d]D_{Ele}[\$/kWh]/\eta_{P\_IT}\times PLF$$

(9) $$CC_{Pre\_RO}[\$]=697\times 0.7\times {\left(Q_{f}[m^{3}/d]/0.9\right)}^{0.78}$$

(10) $$OC_{Pre\_RO}[\$/d]=0.028P_{Pre}[bar]Q_{f}[m^{3}/d]D_{Ele}[\$/kWh]/\eta_{P\_Pre}\times PLF$$

(11) $$CC_{HP\_RO}[\$]=Q_{f}[m^{3}/d](393,000+10,710P_{f,in}[bar]/30,000)$$

(12) $$OC_{HP\_RO}[\$/d]=0.028P_{f,in}[bar]Q_{f}[m^{3}/d]D_{Ele}[\$/kWh]/\eta_{P\_HP}\times PLF$$

(13) $$CC_{BP\_RO}[\$]=(Q_{f}[m^{3}/d]-Q_{p}[m^{3}/d])(393,000+10,710P_{f,in}[bar]/60,000)$$

(14) $$OC_{BP\_RO}[\$/d]=\frac{(0.028(P_{f,in}-P_{f,out})[bar]\eta_{ERD}(Q_{f}-Q_{p})[m^{3}/d]D_{Ele}}{\eta_{P\_BP}}\times PLF$$

(15) $$CC_{ERD\_RO}[\$]=(Q_{f}[m^{3}/d]-Q_{p}[m^{3}/d])(393,000+10,710P_{f,in}[bar]/21,600),$$

where *CC* and *OC* denote the capital cost and operating cost, respectively. *P* and *η* are pressure and efficiency, respectively. The subscripts *IT*, *Pre*, *HP*, *BP*, and *ERD* denote the intake, pre-treatment,HP pump, booster pump, and energy recovery device, respectively. *Q_f_* and *Q_p_* are the feed and permeate flow rates, respectively. Finally, *D_energy_* is the unit electricity price, and *PLF* is the plant load factor.

Assuming that the capital cost of the membrane module is linear to the membrane area, the annualized capital cost of the membrane is calculated as follows [19].

(16) $$CC_{Mem\_RO}[\$]=Area_{Mem\_RO}C_{mem\_RO}[\$/m^{2}],$$

where the subscript *Mem* denotes the membrane, *Area_mem_RO_* is the total RO membrane area, and *C_Mem_RO_* is the unit RO membrane cost.

The total capital cost comprises the direct capital cost and the indirect capital cost. The direct capital cost is the sum of the cost of system equipment and the cost of site development, the latter of which is set to 20% of the equipment cost [15]. The indirect capital cost is set to 30% of the direct capital cost [15]. The total and annual capital costs of the RO process are expressed as follows [12,13,14,15].

(17) $$CC_{Equipment\_RO}[\$]=CC_{IT\_RO}+CC_{Pre\_RO}+CC_{HP\_RO}+CC_{BP\_RO}+CC_{ERD\_RO}+CC_{Mem\_RO}$$

(18) $$CC_{Site\_RO}[\$]=CC_{Equipment\_RO}\times 0.2$$

(19) $$DCC_{RO}[\$]=CC_{Equipment\_RO}+CC_{Site\_RO}$$

(20) $$ICC_{RO}[\$]=DCC_{RO}\times 0.3$$

(21) $$TCC_{RO}[\$]=DCC_{RO}+ICC_{RO}$$

(22) $$ACC_{RO}[\$/y]=TCC_{RO}\frac{i{\left(1+i\right)}^{n}}{{\left(1+i\right)}^{n}-1},$$

where *DCC_RO_* is the direct capital cost, *ICC_RO_* is the indirect capital cost, *TCC_RO_* is the total capital cost, *ACC_RO_* is the annual capital cost, *i* is the interest rate, and *n* is the plant lifetime.

The annual operating cost comprises the annual power cost, annual membrane replacement cost, and other costs (e.g., labor, chemicals, and maintenance). The annual operating costs of the RO process are expressed as follows [12,13,14,15].

(23) $$OC_{Power\_RO}[\$/y]=(OC_{IT\_RO}+OC_{Pre\_RO}+OC_{HP\_RO}+OC_{BP\_RO})\times 365$$

(24) $$OC_{MR\_RO}[\$/y]=CC_{Mem\_RO}\times 0.2$$

(25) $$OC_{etc\_RO}[\$/y]=AOC_{RO}\times 0.3$$

(26) $$AOC_{RO}[\$/y]=OC_{power\_RO}+OC_{MR\_RO}+OC_{etc\_RO},$$

where $OC_{power\_RO}$ is the annual power cost, $OC_{MR\_RO}$ is the annual membrane replacement cost, and $OC_{etc\_RO}$ is the other costs.

Finally, the water cost of the RO process is calculated as follows.

(27) $$WC_{RO}[\$/m^{3}]=(ACC_{RO}+AOC_{RO})/(365\times Q_{P}\times PLF)$$

### 2.2. Forward Osmosis Model

According to the solution-diffusion model, the general water flux (*J_w_*) and salt flux (*J_s_*) equations for FO can be defined as follows.

(28) $$J_{w}=A\left(\pi_{D,b}-\pi_{F,b}\right)$$

(29) $$J_{s}=B\left(C_{D,b}-C_{F,b}\right),$$

where *π_D,b_* is the osmotic pressure at the draw solution side, *π_F,b_* is the osmotic pressure at the feed side, and *C_D,b_* and *C_F,b_* are the concentrations at the draw solution and feed side, respectively. The osmotic pressure directly relates to the concentration of each solution, with the modified van’t Hoff formula.

(30) $$\pi =\frac{NRT}{M_{w}}C,$$

where *N* is the ionization number in the water, *R* is the ideal gas constant, *T* is the temperature, *M_w_* is the molecular weight, and *C* is the salt concentration. That is, the water flux is in an amount proportional to the concentration difference between the two solutions. The measured water flux was much lower than the expected value, due to the effects of ECP and ICP. To make the measurements more accurate, the general water and salt flux equations were modified while considering ECP and ICP. There are two types of ICP and ECP, depending on possible membrane orientations. In the PRO mode, the dilutive ECP is coupled with the concentrative ICP; in the other orientation—namely, the FO mode—the concentrative ECP is coupled with the dilutive ICP [4]. In this study, only the concentrative ECP coupled with the dilutive ICP is considered. For the modeling of concentrative ECP, a mass transfer equation more commonly used by RO was employed, coupled with boundary layer film theory [4].

(31) $$\pi_{F,m}=\pi_{F,b}\exp\left(\frac{J_{w}}{k}\right),$$

where *k* is the mass transfer coefficient for ECP, *π_F,b_* is the osmotic pressure of the feed in the bulk, and *π_F,m_* is the osmotic pressure on the membrane surface. The mass transfer coefficient for ECP, *k*, can correlate with the Sherwood number [4].

(32) $$k=\frac{ShD}{d_{h}}$$

(33) $$Sh=1.85{\left(Re\;Sc\frac{d_{h}}{L}\right)}^{0.33}\;(Re\;\leq \;2100)$$

(34) $$Sh=0.04{Re}^{0.75}{Sc}^{0.33}\;(Re\;>\;2100)$$

To identify flux behavior in the presence of the dilutive ICP, Loeb *et al.* [19] present a simplified governing equation for ICP in asymmetric membranes.

(35) $$K={\left(\left(\frac{1}{J_{w}}\right)\ln\frac{B+A\pi_{D,b}}{B+J_{w}+A\pi_{F,m}}\right)}^{-1}=$$

where *K* is the ICP resistance. In the FO mode, the non-measurable variables *C_F,m_* and *C_D,m_* (*i.e.*, the draw solution concentration at the interface between the active layer and the support layer, respectively) can be calculated by modifying Equations (31) and (35) as follows [19].

(36) $$C_{F,m}=C_{F,b}\exp\left(\frac{J_{w}}{k}\right)$$

(37) $$C_{D,m}=C_{F,m}-\frac{C_{F,m}-C_{D,b}\exp\left(-J_{w}K\right)}{1-\frac{B}{J_{w}}\left[\exp\left(-J_{w}K\right)-1\right]}$$

The effective osmotic pressure can be calculated by using Equation (28) for the feed and draw solution with the calculated concentration. Accordingly, the effective driving force is determined for estimating the water flux. Using *C_D,m_* and *C_F,m_* instead of *C_D,b_* and *C_F,b_*, the water flux (*J_w_*) and salt flux (*J_s_*) equations can be modified as follows.

(38) $$J_{w}=A\left(\frac{N_{D}RT_{D}}{M_{w,D}}\left(C_{F,m}-\frac{C_{F,m}-C_{D,b}\exp\left(-J_{w}K\right)}{1-\frac{B}{J_{w}}\left[\exp\left(-J_{w}K\right)-1\right]}\right)-\frac{N_{F}RT_{F}}{M_{w,F}}C_{F,b}\exp\left(\frac{J_{w}}{k}\right)\right)$$

(39) $$J_{s}=B\left(C_{F,m}-\frac{C_{F,m}-C_{D,b}\exp\left(-J_{w}K\right)}{1-\frac{B}{J_{w}}\left[\exp\left(-J_{w}K\right)-1\right]}-C_{F,b}\exp\left(\frac{J_{w}}{k}\right)\right)$$

*k* can be easily calculated using known experimental conditions, in conjunction with Equation (30). However, it is more complicated to calculate *K*, as it is a function of membrane characteristics that are either unavailable or difficult to determine, given proprietary issues [4]. The water flux equation is very difficult to solve analytically, since the water flux is dependent on itself, given the inclusion of the ECP and ICP terms. To solve the water flux equation, the membrane was divided into small segments and their sizes were chosen so as to be consecutively smaller, until they were sufficiently small that the error of the calculated results was tolerable.

**Figure 2:**
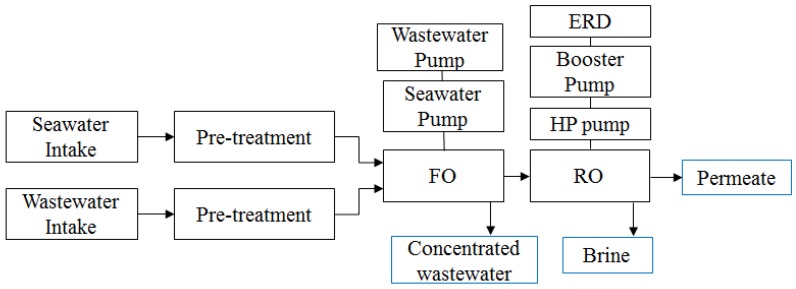
Schematic diagram of FO-RO hybrid system, used to evaluate economic feasibility.

The FO cost model is almost identical to the RO cost model. The FO cost model was developed to evaluate the FO-RO hybrid system, shown as Figure 2.

The FO process consists of four major parts—namely, the seawater and feed water intake, the pre-treatment process, the seawater and treated wastewater circulation pump, and the FO membrane module. The capital and operating costs of the intake, pre-treatment process, seawater and wastewater circulation pump and FO membrane module are expressed as follows.

(40) $$CC_{IT\_S\_FO}[\$]=598\times {\left(Q_{S}[m^{3}/d]/0.9\right)}^{0.78}$$

(41) $$OC_{IT\_S\_FO}[\$/d]=0.028P_{IT\_S}[bar]Q_{S}[m^{3}/d]D_{Ele}[\$/kWh]/\eta_{IT\_S}\times PLF$$

(42) $$CC_{IT\_W\_FO}[\$]=598\times {\left(Q_{W}[m^{3}/d]/0.9\right)}^{0.78}$$

(43) $$OC_{IT\_S\_FO}[\$/d]=0.028P_{IT\_W}[bar]Q_{W}[m^{3}/d]D_{Ele}[\$/kWh]/\eta_{IT\_W}\times PLF$$

(44) $$CC_{Pre\_S\_FO}[\$]=697\times {\left(Q_{S}[m^{3}/d]/0.9\right)}^{0.78}$$

(45) $$OC_{Pre\_S\_FO}[\$/d]=0.028P_{Pre\_S}[bar]Q_{S}[m^{3}/d]D_{Ele}[\$/kWh]/\eta_{Pre\_S}\times PLF$$

(46) $$CC_{Pre\_W}[\$]=697\times {\left(Q_{W}[m^{3}/d]/0.9\right)}^{0.78}$$

(47) $$OC_{Pre\_W\_FO}[\$/d]=0.028P_{Pre\_W}[bar]Q_{W}[m^{3}/d]D_{Ele}[\$/kWh]/\eta_{Pre\_W}\times PLF$$

(48) $$CC_{P\_S\_FO}[\$]=Q_{S}[m^{3}/d](393,000+10,710P_{S}[bar]/30,000)$$

(49) $$OC_{P\_S\_FO}[\$/d]=0.028P_{S}[bar]Q_{S}[m^{3}/d]D_{Ele}[\$/kWh]/\eta_{P\_S}\times PLF$$

(50) $$CC_{P\_W\_FO}[\$]=Q_{W}[m^{3}/d](393,000+10,710P_{W}[bar]/30,000)$$

(51) $$OC_{P\_W\_FO}[\$/d]=0.028P_{W}[bar]Q_{W}[m^{3}/d]D_{Ele}[\$/kWh]/\eta_{P\_W}\times PLF,$$

(52) $$CC_{Mem\_FO}[\$]=Area_{Mem\_FO}C_{mem\_FO}[\$/m^{2}]$$

where the subscripts *S* and *W* denote the seawater and treated wastewater, respectively.

In this study, the feed water of the FO process was assumed to be treated wastewater; therefore the pre-treatment of treated wastewater was set to be the same as for seawater. The annualized capital cost of the FO-RO hybrid system was calculated in the same manner as for the RO process. The total capital cost comprises the direct capital cost and the indirect capital cost. The direct capital cost is the sum of the cost for system equipment and the cost for site development, the latter of which is set at 20% of the equipment cost [13]. The indirect capital cost is set at 30% of the direct capital cost. The total and annual capital costs of FO-RO process are expressed as follows [12,13,14,15].

(53) $$\begin{array}{l} CC_{Equipment\_FO}[\$]=CC_{IT\_S\_FO}+CC_{IT\_W\_FO}+CC_{Pre\_S\_FO}+CC_{Pre\_W\_FO}\\ +CC_{P\_S\_FO}+CC_{P\_W\_FO}+CC_{Mem\_FO} \end{array}$$

(54) $$CC_{Equipment\_FO-RO}[\$]=CC_{Equipment\_RO}+CC_{Equipment\_FO}$$

(55) $$DCC_{FO=RO}[\$]=CC_{Equipment\_FO-RO}+CC_{Site}$$

(56) $$ICC_{FO-RO}[\$]=DCC_{FO-RO}\times 0.3$$

(57) $$TCC_{FO-RO}[\$]=DCC_{FO-RO}+ICC_{FO-RO}$$

(58) $$ACC_{FO-RO}[\$/y]=TCC_{RO-RO}\frac{i{\left(1+i\right)}^{n}}{{\left(1+i\right)}^{n}-1}$$

The annual operating cost comprises the annual power cost, annual membrane replacement cost, and other costs (e.g., labor, chemicals, and maintenance). The annual operating costs of the FO and FO-RO processes are expressed as follows [12,13,14,15].

(59) $$\begin{array}{l} OC_{Power\_FO}[\$/y]=(OC_{IT\_S\_FO}+OC_{IT\_W\_FO}+OC_{Pre\_S\_FO}+OC_{Pre\_W\_FO}\\ +OC_{P\_S\_FO}+OC_{P\_W\_FO})\times 365 \end{array}$$

(60) $$OC_{MR\_FO}[\$/y]=CC_{Mem\_FO}\times 0.2$$

(61) $$OC_{MR\_FO-RO}[\$/y]=OC_{MR\_RO}+OC_{MR\_FO}$$

(62) $$OC_{etc\_FO-RO}[\$/y]=AOC_{FO-RO}\times 0.3$$

(63) $$OC_{Power\_FO-RO}[\$/y]=(OC_{Power\_RO}+OC_{Power\_FO})\times 365$$

(64) $$AOC_{FO-RO}[\$/y]=OC_{power\_FO-RO}+OC_{Mr\_FO-RO}+OC_{etc\_FO-RO}$$

Finally, the water cost of the FO process is calculated as follows.

(65) $$WC_{FO-RO}[\$/m^{3}]=(ACC_{FO-RO}+AOC_{FO-RO})/(365*Q_{p}\times PLF)$$

## 3. Results and Discussion

### 3.1. Performance Simulation of Forward Osmosis Process

The values of the model parameters and operating conditions used in this study for FO process simulations are listed in Table 1 [20,21,22,23,24,25,26,27,28]. We changed the values of *A*, *B*, and *K* to predict the performance of the FO process.

**Table 1:** Parameters and operating conditions for forward osmosis (FO) process simulations.TDS = total dissolved solids.

Parameter	Value
*FO membrane*	
*A*	1.0~4.5 × 10^−12^ m/s-Pa (3.0 × 10^−12^ m/s-Pa)
*B*	1.0~4.5 × 10^−8^ m/s (1.8 × 10^−8^ m/s)
*K*	0.5~7 × 10^5^ s/m (0.96 × 10^5^ s/m)
Membrane area	20 m^2^/module
Module configuration	Spiral wound (seven modules per vessel)
*TDS*	
Seawater	35,000 mg/L
Treated wastewater	200 mg/L
*Flow rate*	
Seawater	100 m^3^/d per vessel
Treated wastewater	150 m^3^/d per vessel

**Figure 3:**
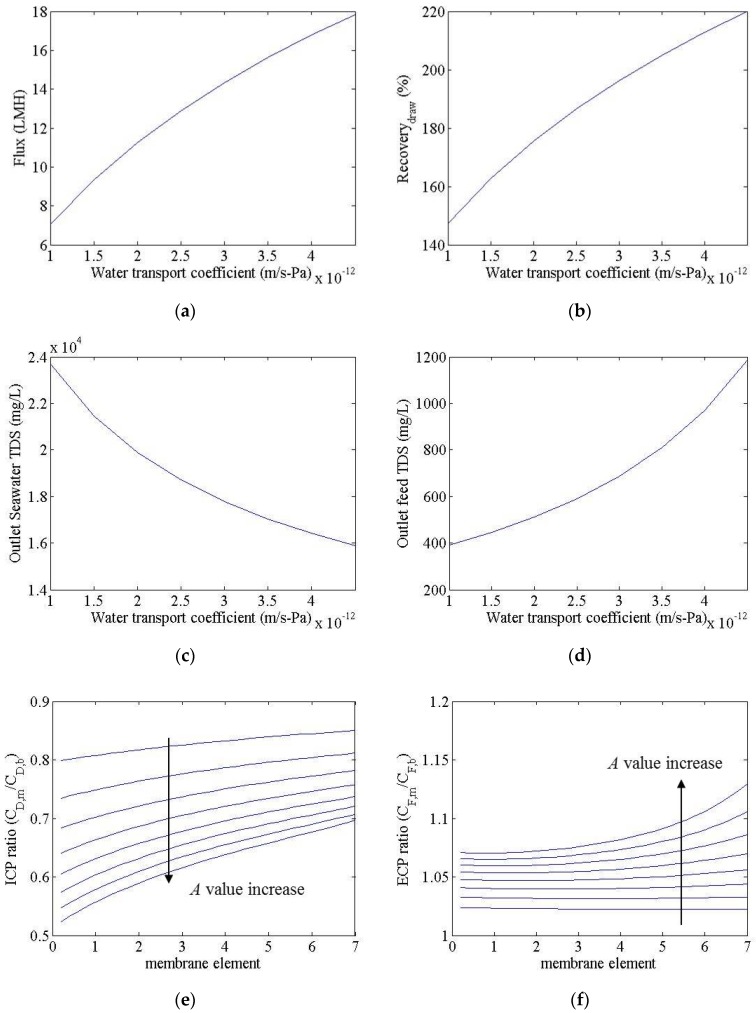
Simulation results for an FO system, with *A* values of 1 × 10^−12^ and 4.5 × 10^−12^ m/s-Pa. (**a**) Flux; (**b**) recovery; (**c**) seawater outflow TDS; (**d**) treated wastewater outflow TDS; (**e**) ICP ratio; (**f**) ECP ratio. (External and internal concentration polarization (ECP and ICP)).

Figure 3 shows the simulation results for an FO process, resulting from a change in the *A* value from 1 × 10^−12^ to 4.5 × 10^−12^ m/s-Pa. In this calculation, the *B* and *K* values were set to 1.8 × 10^−8^ m/s and 0.96 × 10^5^ s/m, respectively. The water flux and recovery went from 7 to 18 LMH and from 150% to 220%, respectively. The total dissolved solids (TDS) of seawater outflow and treated wastewater outflow were calculated as changing from 16,000 to 24,000 mg/L and from 400 to 1200 mg/L, respectively. In this study, the recovery of the FO process is defined as the ratio of the seawater inflow rate to the seawater outflow rate. The water flux and recovery increased in accordance with an increased *A* value; however, unlike in the RO process, the water flux here was not in direct linear proportion to the *A* value. In the FO process the negative contribution of ECP and ICP to the water flux increased as the water flux increased, as shown in Figure 3e,f, since water flux is self-limited. This suggests that increasing the water transport coefficient will provide diminishing increases in water flux. The ECP ratio is the ratio of treated wastewater concentration to the membrane surface and treated wastewater concentration in the bulk solution. The ICP ratio, meanwhile, is the ratio of seawater concentration in the membrane support layer to the seawater concentration in the bulk solution. The ECP and ICP ratios decreased in line with an increasing membrane module in a vessel; this can be attributed to an increased concentration of treated wastewater and a reduced seawater concentration, resulting in a decrease in the net driving force. The seawater concentration in the product decreased as the *A* value increased; this characteristic can help reduce the energy consumption of the RO process within the FO-RO hybrid process.

**Figure 4:**
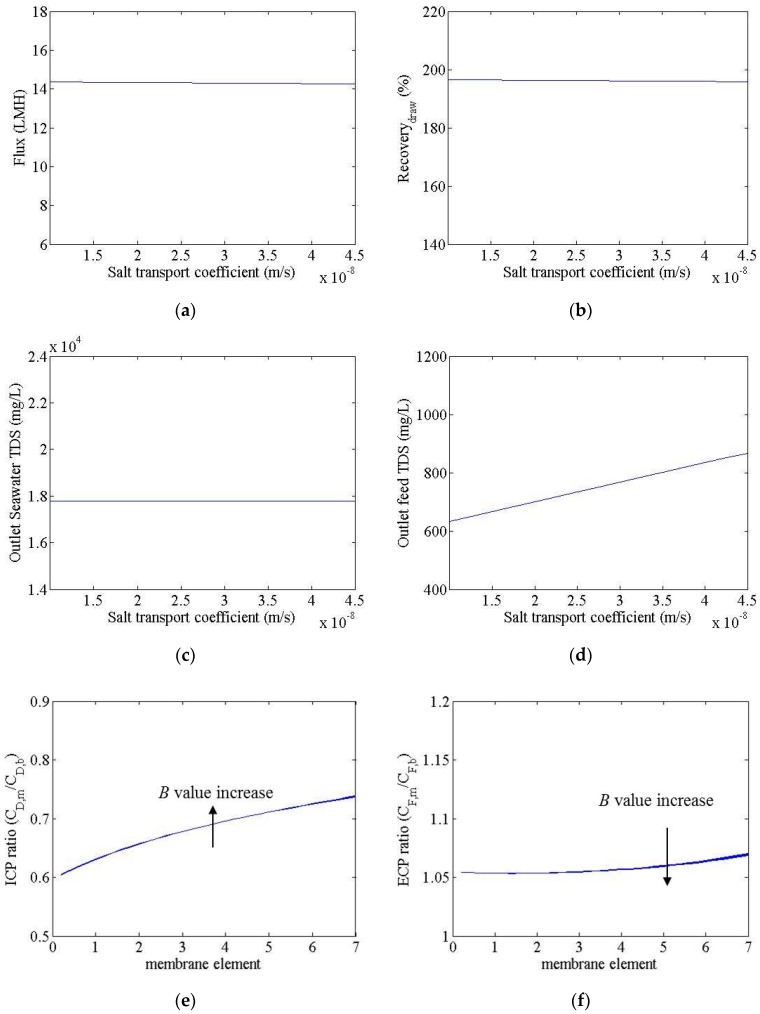
Simulation results for an FO system, with *B* values of 1 × 10^−8^ and 4.5 × 10^−8^ m/s. (**a**) Flux(**b**) recovery; (**c**) seawater outflow TDS; (**d**) treated wastewater outflow TDS; (**e**) ICP ratio; (**f**) ECP ratio.

Figure 4 shows the simulation results for an FO process with a change in the *B* value from 1.0 × 10^–8^ to 4.5 × 10^–8^ m/s. In this calculation, the *A* and *K* values were set to 3.0 × 10^–12^ m/s-Pa and 0.96 × 10^5^ s/m, respectively. The water flux, recovery, seawater outflow TDS, and ICP and ECP ratios showed little change, despite an increased *B* value. Only the feed water outflow TDS increased, from 620 to 860 mg/L; this is because the seawater concentration on the membrane surface was significantly reduced relative to the bulk solution concentration, due to ICP. In the first module, the seawater concentration decreased from 35,000 to 21,000 mg/L.

**Figure 5:**
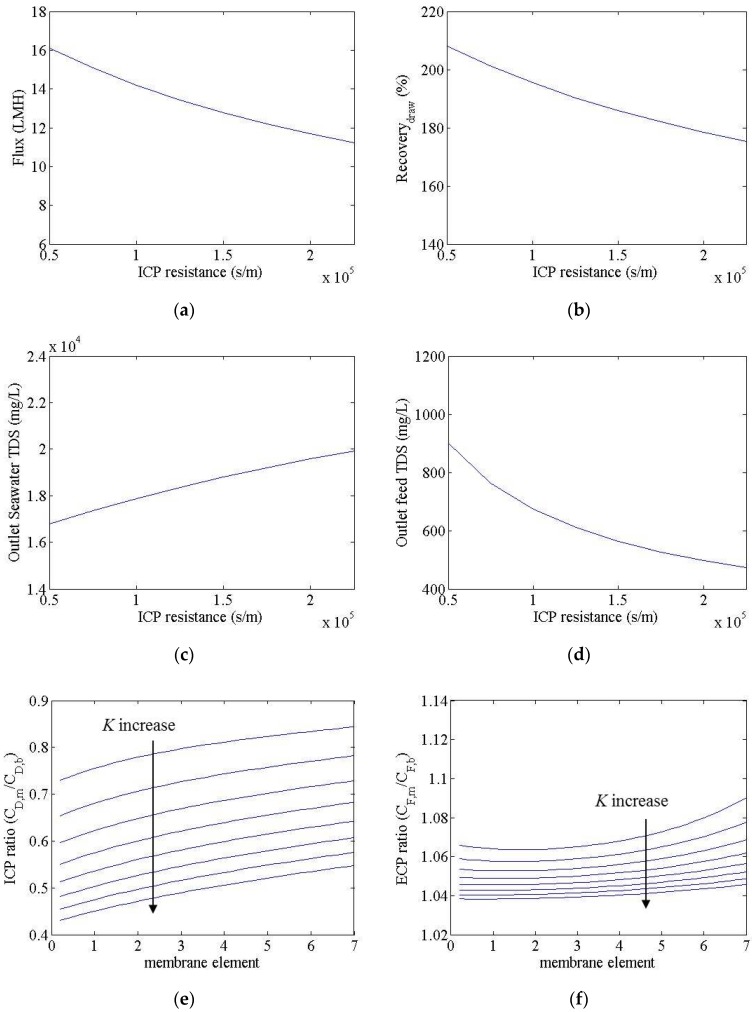
Simulation results for an FO system, with *K* values of 0.5 × 10^5^ and 2.25 × 10^5^ s/m. (**a**) Flux; (**b**) recovery; (**c**) seawater outflow TDS; (**d**) treated wastewater outflow TDS; (**e**) ICP ratio; (**f**) ECP ratio.

Figure 5 shows the simulation results for an FO process with a change in the *K* value from 0.5 × 10^5^ to 2.25 × 10^5^ s/m. Here, the *A* and *B* values were set to 3.0 × 10^−12^ m/s-Pa and 1.8 × 10^8^ m/s, respectively. The water flux and recovery changed from 11 to 16 LMH and from 175% to 210%, respectively. Additionally, the TDS of the seawater outflow and treated wastewater outflow changed from 17,000 to 20,000 mg/L and from 500 to 900 mg/L, respectively. The water flux and recovery value decreased as the *K* value increased; however, the TDS of seawater outflow decreased as the *K* value increased. The seawater concentration in the support layer of the FO membrane decreased, while the treated wastewater concentration on the FO membrane surface decreased as the *K* value increased. However, the decrement of the seawater concentration in the support layer was much higher than that of the treated wastewater concentration on the membrane surface, as shown in Figure 5e,f. This was because the water flux decreased as the *K* value increased.

### 3.2. Cost Estimation of Reverse Osmosis and Forward Osmosis–Reverse Osmosis Hybrid Process

To investigate the effect of FO-RO hybridization, evaluations of economic feasibility were undertaken with an FO-RO hybrid desalination system, and 100,000 m^3^/d RO. The values of the model parameters and operating conditions for RO and the FO-RO system used in this study are listed in Table 2. The intake pressure is set to 5.0 bar and pretreatment pressure and recovery are set to 1.0 bar and 90% respectively for RO process. In the FO-RO hybrid process the seawater intake pressure is set to 5.0 bar and treated wastewater intake pressure is set to 1.0 bar. Also, the pressure and recovery of pretreatment for seawater are set to 1.0 bar and 90% and pressure and recovery of pretreatment for treated wastewater are set to 2.0 bar and 90% respectively. In the FO-RO hybrid process the treated wastewater flow rate is set to 1.5 times higher than seawater flow rate.

**Table 2:** Process parameters and operating conditions for economic evaluations.

Parameter	Value	Parameter	Value
*RO*		*FO-RO*	
Membrane area	40 m^2^	Membrane area (FO/RO)	40 m^2^/40 m^2^
Permeate flux	15 LMH	Permeate flux (FO/FO)	15 LMH/15 LMH
Recovery	40%	Recovery (FO/RO)	180%/60%
Seawater TDS	35,000 mg/L	Seawater TDS	35,000 mg/L
		Treated wastewater TDS	200 mg/L
*Efficiency*		*Efficiency*	
Pump	75%	Pump	75%
ERD	95%	ERD	95%
*Cost*		*Cost*	
Electricity bill	USD 0.1/kWh	Electricity bill	USD 0.1/kWh
Membrane cost	USD 40/m^2^	Membrane cost (FO-RO)	USD 40/USD 40 m^2^
System load factor	0.91	System load factor	0.91
Interest rate	0.08	Interest rate	0.08
System life	20 years	System life	20 years

Table 3 shows the calculation results for an RO system, using the parameters and operating conditions listed in Table 2.

**Table 3:** Calculation results for a 100,000 m^3^/d RO system.

Parameter	Value
*Energy Consumption*	
Intake	2140 kW
Pre-treatment	430 kW
HP pump	8900 kW
Booster pump	1130 kW
Specific energy	2.92 kWh/m^3^
*Water Quality*	
Permeate TDS	220 mg/L
Brine TDS	58,200 mg/L
*Capital Cost*	
Intake	USD 10,532,000
Pre-treatment	USD 12,287,000
HP pump	USD 25,683,000
Booster pump	USD 9,348,000
Energy recovery device	USD 6,918,000
Membrane	USD 11,121,000
Site	USD 13,000,000
Direct capital cost	USD 77,254,000
Indirect capital cost	USD 23,257,000
Capital cost	USD 100,511,000
Annual capital cost	USD 10,265,000/year
*Operating Cost*	
Power cost	USD 9,700,000/year
Membrane replacement	USD 2,240,000/year
Labor, chemicals, and maintenance	USD 5,110,000/year
Annual operating cost	USD 17,050,000/year
*Water cost*	
Water cost	USD 0.75/m^3^

The water cost of the RO system was calculated as USD 0.79/m^3^. The cost of the RO system construction constituted 38% of the total water cost. The cost of energy, membrane replacement, and labor, chemicals, and maintenance for the RO system constituted 36%, 8%, and 18% of the total water cost, respectively. It is impossible to predict with great accuracy the cost of water for an RO system, as there are many factors that can influence the cost. However, in this study, the main factors that affect the cost of water were adequately applied, and the results suggested reasonable costs compared to those in the Global Water Initiative 2015 report.

Four parameters were used to evaluate the economic feasibility of a 100,000 m^3^/d FO-RO hybrid desalination system. The flux of the FO process, recovery of the FO process, FO membrane cost, and electricity cost were found to be the parameters with the greatest effect on water cost. First, we made a change to the flux of the FO process; the other factors namely, recovery of the FO process, FO membrane, and electricity cost were fixed. The flux changed from 10 to 20 LMH. The recovery, membrane, and electricity cost were set to 180%, USD 40/m^3^, and USD 0.1/kWh, respectively. (The other model parameters and operating conditions were as listed in Table 2.) Next, we made changes to the recovery of the FO process, while the other factors were fixed: the recovery rate was changed from 120% to 240%, while the flux, membrane, and electricity cost were fixed to 15 LMH, USD 40/m^2^, and USD 0.1/kWh, respectively. Thereafter, we made changes to the FO membrane cost, from USD 30 to USD 80/m^2^, while the flux, recovery, and electricity cost were fixed to 15 LMH, 180%, and USD 0.1/kWh, respectively. Finally, we made changes to the electricity cost, from USD 0.05 to USD 0.25/kWh, while the flux, recovery, and FO membrane cost were fixed to 15 LMH, 180%, and USD 40/m^2^, respectively.

**Figure 6:**
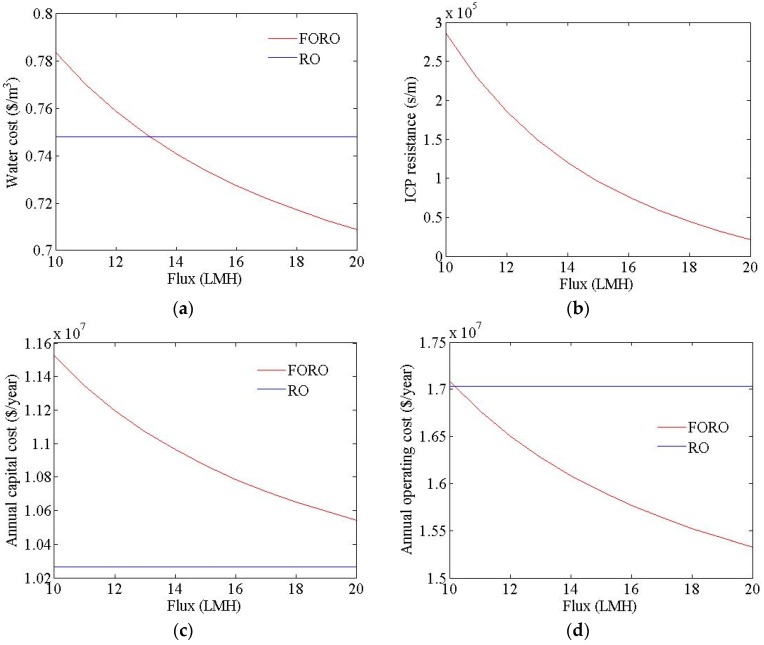

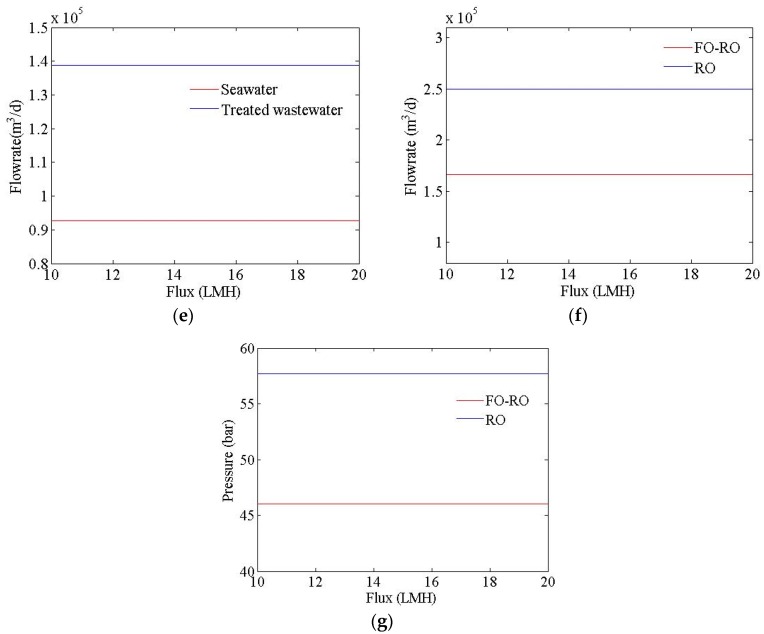
Cost simulation results of FO-RO hybrid system at different permeate flux values. (**a**) Water cost; (**b**) ICP resistance; (**c**) annual capital cost; (**d**) annual operating cost; (**e**) flow rate of the seawater and treated wastewater feed to the FO process; (**f**) flow rate of the seawater feed to the RO process; (**g**) RO feed pressure.

Figure 6 shows the cost estimation results for an FO-RO hybrid system at different FO flux values. In the FO process, the ICP resistance was adjusted to change the water flux, as shown in Figure 6b. The water cost of the hybrid system was found to range from USD 0.71 to USD 0.78/m^3^, in line with changes to the water flux. The specific energy, permeate TDS, and brine TDS of the FO-RO hybrid system were calculated as 2.26 kWh/m^3^, 163 mg/L, and 48,300 mg/L, respectively. The flow rate of the seawater and treated wastewater feed to the FO process in FO-RO hybrid system were calculated as 92,592 m^3^/d and 138,888 m^3^/d respectively. The flow rate of the seawater feed to the RO process in FO-RO hybrid system was calculated as 166,666 m^3^/d. The feed pressure of the RO process in the FO-RO hybrid system was calculated as 46.0 bar. The water cost of the hybrid system decreased with increasing water flux, given the reduced size of the required FO membrane area; therefore, both the capital cost and the operating cost decreased with increasing water flux, as shown in Figure 6c,d. In this calculation, the seawater outflow TDS was 19,400 mg/L; this constituted 55.4% of the seawater inflow TDS, regardless of water flux, as the recovery of the FO process was fixed to 180%. This reduced the feed pressure of the RO process from 57.6 bar to 46.0 bar. Therefore, the energy consumption of the hybrid system was lower than that of the RO system (2.92 *vs.* 2.26 kWh/m^3^). The water cost of the RO system was USD 0.75/m^3^. To make the FO-RO hybrid system more price-competitive than the RO system, the water flux under these simulation conditions should exceed 13.5 LMH. The permeate and brine TDS of the hybrid system were much lower than those of the RO system, since the RO feed TDS of the hybrid system was much lower than that of the RO system.

**Figure 7:**
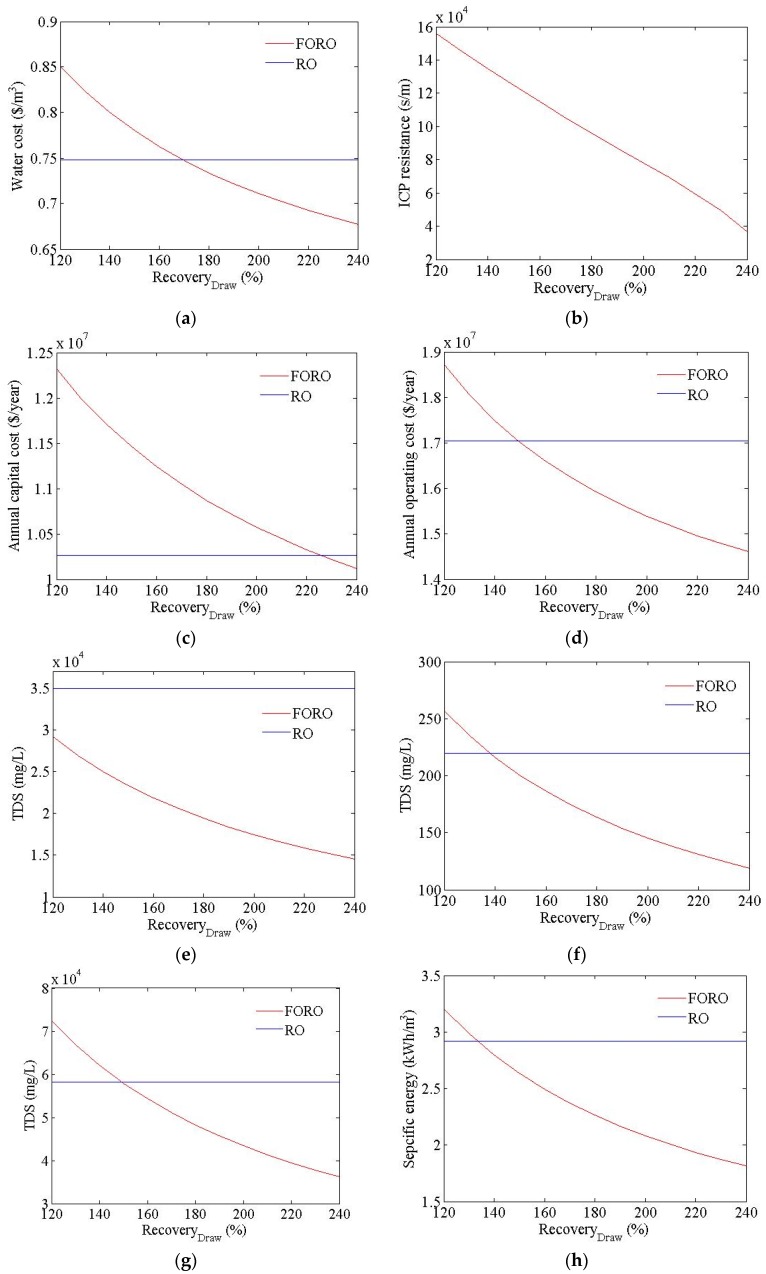

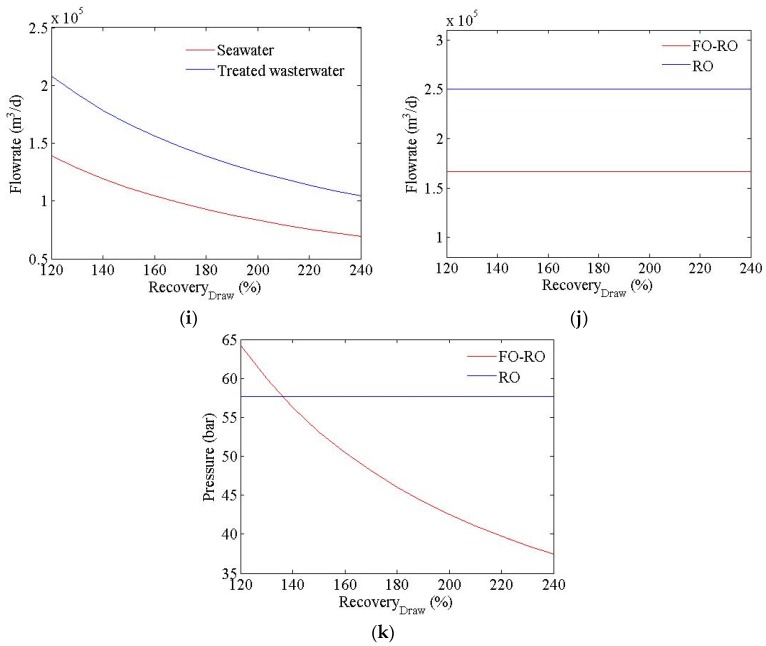
Cost simulation results of FO-RO hybrid system at different rates of recovery in the FO process. (**a**) Water cost; (**b**) ICP resistance; (**c**) annual capital cost; (**d**) annual operating cost; (**e**) RO feed TDS; (**f**) permeate TDS; (**g**) brine TDS; (**h**) specific energy; (**i**) flow rate of the seawater and treated wastewater feed to the FO process; (**j**) flow rate of the seawater feed to the RO process; (**k**) RO feed pressure.

Figure 7 shows the cost estimation results for the FO-RO hybrid system at different rates of recovery within the FO process. In the FO process, the ICP resistance is also adjusted to alter the recovery FO process, as shown as Figure 6b. The water cost of the hybrid system was calculated as ranging from USD 0.71 to USD 0.87/m^3^, in line with changes in recovery. The flow rate of the seawater and treated wastewater feed to the FO process in the FO-RO hybrid system were calculated as ranging from 69,444 m^3^/d to 136,888 m^3^/d and from 104,166 m^3^/d to 208,333 m^3^/d respectively. The flow rate of the seawater feed to the RO process in FO-RO hybrid system was calculated as 166,666 m^3^/d. The feed pressure of the RO process in the FO-RO hybrid system was calculated as ranging from 37.4 bar to 64.3 bar. The water cost of the hybrid system decreased with the increased recovery of the FO process, since the seawater and treated wastewater flow rate, energy consumption, and required membrane area all decreased. Therefore, the capital cost and operating cost decreased with the increased recovery of the FO process, as seen in Figure 7c,d. In this calculation, the seawater outflow TDS changed from 14,600 to 29,200 mg/L and the energy consumption changed from 1.82 to 3.20 kWh/m^3^, as shown in Figure 7d,h. The TDS of the permeate and brine decreased with the increased recovery of the FO process, since the feed water TDS of the RO process had decreased. The FO-RO hybrid system can be price-competitive relative to an RO system. For the FO-RO hybrid system to be more price-competitive than an RO system, under these simulation conditions, the recovery rate should exceed 170%.

**Figure 8:**
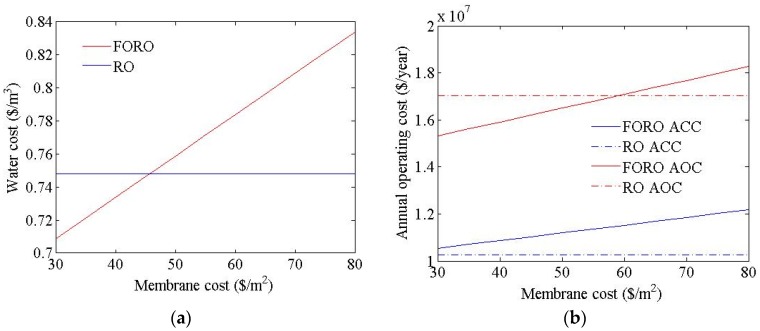
Cost simulation results of the FO-RO hybrid system at different FO membrane cost values. (**a**) Water cost; (**b**) ICP resistance.

Figure 8 shows the cost estimation results for an FO-RO hybrid system with various FO membrane cost values. The water cost of the hybrid system was found to range from USD 0.71 to USD 0.84/m^3^, in line with changes to the FO membrane cost. The flow rate of the seawater and treated wastewater feed to the FO process in FO-RO hybrid system were calculated as 92,592 m^3^/d and 138,888 m^3^/d respectively. The flow rate of the seawater feed to the RO process in FO-RO hybrid system was calculated as 166,666 m^3^/d. The feed pressure of the RO process in the FO-RO hybrid system was calculated as 46.0 bar. For the FO-RO hybrid system to be price-competitive relative to an RO system, in these simulation conditions, the FO membrane cost should be no higher than USD 45/m^2^.

**Figure 9:**
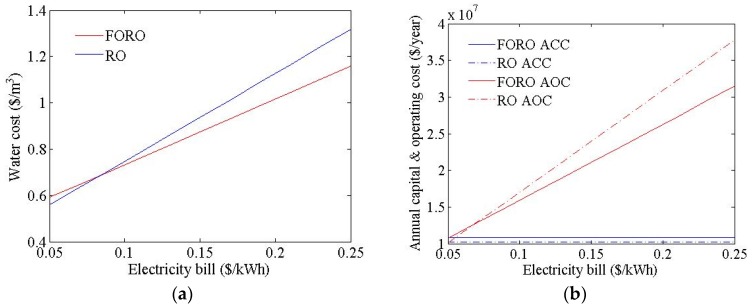
Cost simulation results of the FO-RO hybrid system at different electricity cost values. (**a**) Water cost; (**b**) ICP resistance.

Figure 9 shows the cost estimation results of the RO and FO-RO hybrid systems, with different electricity cost values. The water cost is highly sensitive to the electricity cost: the water cost of the hybrid system was found to range from USD 0.59 to USD 1.16/m^3^, in line with changes to the electricity cost. The flow rate of the seawater and treated wastewater feed to the FO process in the FO-RO hybrid system were calculated as 92,592 m^3^/d and 138,888 m^3^/d respectively. The flow rate of the seawater feed to the RO process in the FO-RO hybrid system was calculated as 166,666 m^3^/d. The feed pressure of the RO process in the FO-RO hybrid system was calculated as 46.0 bar, same as before. The electricity cost is only coupled with the power cost for systems; therefore, the annual capital cost of the hybrid system is constant, regardless of the electricity cost. For the FO-RO hybrid system to be price-competitive relative to an RO system, under these simulation conditions, the electricity cost should exceed USD 0.08/kWh.

## 4. Conclusions

This study undertook evaluations of the performance and economic feasibility of RO and FO-RO hybrid systems, to propose guidelines by which an FO-RO system could be made price-competitive. These evaluations were conducted through the use of a simple model. The following conclusions can be drawn from this work.

- In the FO process of an FO-RO hybrid system, membrane parameters such as the water transport coefficient and ICP resistance are very important factors that affect performance. On the other hand, the effect of the salt transport coefficient does not seem to be large.
- The flux and recovery of the FO process, the FO membrane, and the electricity cost were found to be very important factors that influence the water cost of an FO-RO hybrid system.
- An FO-RO hybrid system can be price-competitive relative to an RO system when its recovery rate is very high, the flux and membrane cost of the FO are similar to those of RO, and the electricity cost is expensive.
- The most important consideration in commercializing the FO process is enhancing performance in terms of flux and the recovery of FO membranes.
- The use of a simple model can help us understand the characteristics of the FO process.
- Further research is required to determine the optimal FO and RO configurations for use in various applications.
